# Residential Proximity to Freeways and Autism in the CHARGE Study

**DOI:** 10.1289/ehp.1002835

**Published:** 2010-12-16

**Authors:** Heather E. Volk, Irva Hertz-Picciotto, Lora Delwiche, Fred Lurmann, Rob McConnell

**Affiliations:** 1 Departments of Preventive Medicine and Pediatrics, Zilkha Neurogenetic Institute, Keck School of Medicine, Children’s Hospital Los Angeles, University of Southern California, Los Angeles, California, USA; 2 Department of Public Health Sciences, University of California–Davis, Davis, California, USA; 3 Sonoma Technology Inc., Petaluma, California, USA; 4 Department of Preventive Medicine, Keck School of Medicine, University of Southern California, Los Angeles, California, USA

**Keywords:** autism, epidemiology, gene-environment interaction, roadway proximity, traffic emissions

## Abstract

**Background:**

Little is known about environmental causes and contributing factors for autism. Basic science and epidemiologic research suggest that oxidative stress and inflammation may play a role in disease development. Traffic-related air pollution, a common exposure with established effects on these pathways, contains substances found to have adverse prenatal effects.

**Objectives:**

We examined the association between autism and proximity of residence to freeways and major roadways during pregnancy and near the time of delivery, as a surrogate for air pollution exposure.

**Methods:**

Data were from 304 autism cases and 259 typically developing controls enrolled in the Childhood Autism Risks from Genetics and the Environment (CHARGE) study. The mother’s address recorded on the birth certificate and trimester-specific addresses derived from a residential history obtained by questionnaire were geocoded, and measures of distance to freeways and major roads were calculated using ArcGIS software. Logistic regression models compared residential proximity to freeways and major roads for autism cases and typically developing controls.

**Results:**

Adjusting for sociodemographic factors and maternal smoking, maternal residence at the time of delivery was more likely be near a freeway (≤ 309 m) for cases than for controls [odds ratio (OR) = 1.86; 95% confidence interval (CI), 1.04–3.45]. Autism was also associated with residential proximity to a freeway during the third trimester (OR = 2.22; CI, 1.16–4.42). After adjustment for socioeconomic and sociodemographic characteristics, these associations were unchanged. Living near other major roads at birth was not associated with autism.

**Conclusions:**

Living near a freeway was associated with autism. Examination of associations with measured air pollutants is needed.

Autism is a developmental disorder characterized by significant deficits in social interaction and communication, accompanied by repetitive behaviors (American Psychiatric Association 2000). Data from family and twin studies have long supported the role of genetics in autism etiology (Abrahams and Geschwind 2008; Muhle et al. 2004). Results from linkage, copy number variation, and genomewide association studies further support the importance of genetic risk in this disease (Abrahams and Geschwind 2008; Ma et al. 2009; Wang et al. 2009). Over the last 10 years, the prevalence of diagnoses of autism, and all autism spectrum disorders, has increased (Centers for Disease Control and Prevention 2007a, 2007b, 2009). Although changes in diagnostic criteria and improved ascertainment have been thought to contribute to this increase, recent reports suggest that these factors may not fully explain the rising incidence of autism spectrum disorders (Hertz-Picciotto and Delwiche 2009; King and Bearman 2009). Therefore, it is likely that environmental factors may augment the strong genetic risks implicated in autism etiology.

Air pollution exposure during pregnancy has been reported to have physical and developmental effects on the fetus. High levels of air pollution, including carbon monoxide, nitrogen dioxide, and ambient particulate matter (PM), have been associated with very low and low birth weight, preterm birth, and infant mortality (Currie et al. 2009; Ritz and Yu 1999). Specific pollutants, including ozone, sulfur dioxide, PM, and carbon monoxide, have also been associated with significant differences in biparietal diameter and head circumference measured both during pregnancy and at birth (Hansen et al. 2008; Vassilev et al. 2001). Maternal exposure to polycyclic aromatic hydrocarbons (PAHs) during pregnancy has been associated with impaired cortical function and cognitive developmental delay (Bocskay et al. 2005; Perera et al. 2003, 2004, 2006, 2007).

Exposure to air pollution and its components, not only in the prenatal period but also in early postnatal life, has been linked to poor developmental outcomes as well. A recent epidemiologic study reported that use of gas appliances and increased nitrogen dioxide in the home during the first 3 months of life are associated with decreased cognitive test scores and increased inattention at 4 years of age (Morales et al. 2009). In a separate study, Suglia et al. (2008) estimated lifetime residential exposure to black carbon, a proxy for traffic-related PM, among 8- to 11-year-old children and reported decreased performance on intelligence and memory tasks with increasing black carbon levels. Additionally, autism has been associated with estimated regional concentrations of hazardous air pollutants, including arsenic and nickel, and with diesel PM exposure in early childhood (Windham et al. 2006).

Thus, an emerging literature suggests that near roadways, traffic-related air pollutants, possibly influenced by specific components such as PM or PAHs, affect neurodevelopment. However, the role of timing for this exposure during pregnancy or early life is not clear, nor has the relationship between traffic-related air pollutants and autism been tested. In this study, we examined the relationship between autism and traffic proximity (a marker of traffic-related air pollution) during the prenatal period and at the time of birth.

## Materials and Methods

We used data from 304 autism cases and 259 typically developing general-population controls from the Childhood Autism Risks from Genetics and the Environment (CHARGE) study, a population-based case–control study of preschool children. The study design is described in detail elsewhere (Hertz-Picciotto et al. 2006). Briefly, CHARGE subjects were between 24 and 60 months of age at the time of recruitment, which occurred during 2003–2009; lived with at least one English- or Spanish-speaking biological parent; were born in California; and resided in one of the study catchment areas at the time of enrollment. Recruitment was facilitated by the California Department of Developmental Services (DDS) and the regional centers with which they contract to coordinate services for persons with autism and other developmental disabilities. Population-based controls were recruited from the sampling frame of birth files from the State of California and were frequency matched by sex, age, and broad geographic area to the autism cases. All births were between 1997 and 2006.

Each participating family was evaluated in person. Children with a DDS diagnosis of autism were evaluated using the Autism Diagnostic Observation Schedules (ADOS), and parents were administered the Autism Diagnostic Interview–Revised (ADI-R) (Le Couteur et al. 2003; Lord et al. 2003). Children with a diagnosed developmental delay and general population controls were given the Social Communication Questionnaire (SCQ) to screen for the presence of autistic features (Rutter et al. 2003). If the SCQ score was ≥ 15, the ADOS was then administered to the child and the ADI-R to the parent. In our study, autism cases were children with a diagnosis of autism from both the ADOS and the ADI-R. All children were also assessed using the Mullen Scales of Early Learning and the Vineland Adaptive Behavior Scales to collect information on motor skills, language, socialization, and daily living skills (Mullen 1995; Sparrow et al. 1984). Controls were children sampled from the general population with typical development, defined as having received a score ≤ 15 on the SCQ and who scored in the normal range on the Mullen Scales of Early Learning and Vineland Adaptive Behavior Scales, thereby showing no evidence of other types of delay (cognitive or adaptive).

Parents were also interviewed extensively to evaluate household exposures and demographic and medical information and to assess reproductive, occupational, and residential histories. The residential history captured addresses and corresponding dates the mother and child lived at each location beginning 3 months before conception and extending to the most recent place of residence. Further details about the collection of clinical and exposure data have been previously reported (Hertz-Picciotto et al. 2006).

We examined associations of autism with traffic-related pollutant exposure using two broad proxies: distance to the nearest freeway and distance to the nearest major road. In accord with our previous research, a freeway was defined as a state highway or interstate highway (Gauderman et al. 2007). A major road was defined as a state highway, interstate highway, or major arterial (McConnell et al. 2006). Mother’s residential address at birth, as recorded on the birth certificate, was geocoded, and distances to the nearest interstate highway, state highway, and major arterial road were estimated based on the shortest distance from the residence to the middle of the nearest side of each of the three road types using ArcGIS software (version 9.2; Environmental Systems Research Institute Inc., Redlands, CA). For each subject, freeway distance was then assigned as the shorter of the distances from the birth residence to a state or interstate highway. Similarly, major road distance was assigned as the shortest of the three distances: from a state highway, interstate highway, or major arterial. Under these definitions, it was possible for freeway and major road distances to be the same should the same road type (e.g., state highway) provide the shortest distance measure for a given address. For freeway and major road distances, we examined the distribution of values among the 563 subjects in our study and determined exposure cut points based on the top 10%, next 15%, and subsequent 25% of distance values for freeways and for major roads. The remaining 50% served as a reference category in each analysis.

Information from the residential history was used to estimate exposure to residential traffic during the first, second, and third trimesters of gestation for a subset of subjects with complete data (*n* = 485; 257 cases and 228 controls). We determined the conception date for each child using gestational age from ultrasound measurements or the date of last menstrual period, as determined from prenatal records. Then we calculated dates corresponding to each trimester and selected the appropriate address from the residential history. If more than one address fell into a trimester, we chose the address where the subjects had spent the most time. Addresses were geocoded and distances estimated as described above.

We used logistic regression to estimate the association between distance to the nearest freeway or major road and autism. Pertinent covariates were included in the model to adjust for potential confounding due to sociodemographic or lifestyle characteristics. Specifically, we included child’s sex and ethnicity, maximum education level of the parents, maternal age, gestational age at birth, and maternal smoking during pregnancy. We obtained 95% confidence intervals (CIs) as measures of precision and determined statistical significance using an alpha level of 0.05.

## Results

### Description of sample

The study population was 84% male, and most participants were Caucasian (51%) or Hispanic (29%). We found no significant differences between cases and controls for any demographic or socioeconomic variables examined (Table 1). For most participants, geocoded birth certificate addresses (mother’s residence at delivery) indicated that residences at birth were concentrated in the areas around Sacramento, Los Angeles, and the San Francisco East and North Bay.

### Distance to freeway

We examined the distribution of distance from the nearest freeway among subjects in our study and determined exposure cut-points to define the closest 10% (< 309 m), the next 15% (309–647 m), and the next 25% (647–1,419 m) as exposure groups. The remaining 50% (> 1,419 m) served as the reference group in our analysis. Living within 309 m of a freeway at birth was associated with autism [odds ratio (OR) = 1.86; 95% CI, 1.04–3.45]. This association was not altered by adjustment for child sex or ethnicity, maximum education in the home, maternal age, or maternal smoking during pregnancy (Table 2). When we categorized our distance measure into deciles, only the top 10%, corresponding to the < 309-m category, showed evidence of an increased autism risk compared with those living farthest from the freeway (lowest decile, > 5,150 m; unadjusted OR = 2.48; 95% CI, 1.17–5.39).

Among the subset of subjects with available residential history data, measures for distance to the freeway were highly correlated across trimesters, reflecting the limited number of subjects who changed residence during pregnancy (*n* = 17 between first and second, 13 between second and third, 30 between first and third). In each trimester, living closest to the freeway (< 309 vs. > 1,419 m) was associated with autism, but the OR reached statistical significance only during the third trimester (adjusted OR = 1.96; 95% CI, 1.01–3.93). Effect estimates for the first and second trimesters were slightly lower in magnitude (first trimester: adjusted OR = 1.66; 95% CI, 0.91–3.10; second trimester: adjusted OR = 1.65; 95% CI, 0.85–3.28). After restricting the sample with birth certificate addresses to those with residential history data for all three trimesters (*n* = 485; 257 cases and 228 controls), the OR for autism was more than doubled among those living within 309 m of a freeway versus > 1,419 m (adjusted OR = 2.22; 95% CI, 1.16–4.42), consistent with a late-pregnancy or early-life effect.

### Distance to major road

The distribution of distance from a major road among subjects in our study was reflected in exposure cut-points corresponding to ≤ 42 m (the closest 10%), 42–96 m (subsequent 15%), and 96–209 m (next 25%) as exposure groups. The remaining 50% (> 209 m) served as the reference group in our analysis. We found no consistent pattern of association of autism with proximity to a major road, and results were changed only slightly after adjusting for distance to the freeway (Table 3). Inclusion of child sex or ethnicity, maximum education in home, maternal age, or prenatal smoking in the model did not alter these associations. Results were similar for the three trimesters.

## Discussion

We observed an increased risk of autism among the 10% of children living within 309 m of a freeway around the time of birth. Our findings appeared to be limited to only this group because analysis of further distances did not demonstrate associations. Analysis of trimester-specific residential information yielded associations of roughly similar magnitude, although only the effects for the third trimester and at birth reached statistical significance. The high correlations across trimesters, and lack of analysis of postnatal residences, imply that we cannot precisely define a potentially critical window.

The association of autism with proximity to freeway, and not to major road, may be related to the larger volume of traffic and concentrations of pollutants observed near freeways. In Los Angeles, for example, some freeways have more than 300,000 vehicles daily and high concentrations of traffic-related pollutants with steep gradients extending several hundred meters from the traffic corridor (Caltrans 2008; Zhu et al. 2002, 2006). Specifically, studies measuring concentration and size distribution of ultrafine PM near a major California freeway demonstrate that the PM is high nearest the freeway and becomes closer to background levels at distances ≥ 300 m (Zhu et al. 2002). Thus, our findings are consistent with the relationship between freeway proximity and PM exposures in California. Our study did not find evidence of associations with residential proximity beyond the 300-m range, and we currently lack adequate sample size to estimate the effect of living in even closer proximity to the freeway (< 100 m) where high concentrations of PM have been detected. To examine the effects of proximity at closer distances to major roadways, we estimated autism risk among subjects living within 96 m (the top quartile of exposure vs. > 96 m) and among those living within 300 m (corresponding to the region of highest exposure vs. > 300 m) and found slightly elevated non-statistically significant risks (within 96 m: OR = 1.17; 95% CI, 0.80–1.72; within 300 m: OR = 1.19; 95% CI, 0.84–1.68).

The traffic volumes on the classes of other major roadways used in this analysis are likely to be highly variable across California, so exposure to traffic-related pollutants on the spatial scale of interest may be less well classified by residential proximity to other major roadways than by proximity to freeways. For example, we found that the average distance to a freeway among subjects living in the second major road exposure group (42–96 m), with slightly increased risk of autism, was much shorter (mean ± SD = 1,481 ± 1,761 m) than in other major road categories (major road < 42 m, 2,643 ± 2,245 m to freeway; major road 96–209 m, 1,917 ± 3,946 m). Residential traffic proximity has been associated with childhood asthma and lung function growth in previous studies we have conducted in Southern California, and some of these associations have been restricted to freeway proximity or traffic modeled from freeway traffic volume (Gauderman et al. 2005, 2007; McConnell et al. 2006, 2010).

We found little evidence of confounding by the socioeconomic and sociodemographic characteristics included in this analysis. We hypothesized these confounders *a priori* based on literature reporting increased autism rates in higher socioeconomic areas, whereas lower socioeconomic areas are more likely to have higher levels of air pollutants (Sexton et al. 1993). In our study, we observed no difference in level of education in the home among autism cases and controls, and adjusting for these factors had little effect on the traffic and autism association, suggesting that our results were not biased by such factors. In California, clusters of autism tend to have higher levels of parental education, and in countries with highly variable access to health care, diagnosed cases of autism tend to be in families with higher socioeconomic status than the general population; at the same time, controls that participate in studies are almost always of higher socioeconomic status than nonparticipants (Van Meter et al. 2010).

To date, little research has examined the association of air pollutants and autism. Using the U.S. Environmental Protection Agency Hazardous Air Pollutants monitoring network, Windham et al. (2006) identified an increased autism risk with modeled estimates of regional census tract ambient exposure to diesel exhaust particles, as well as metals (mercury, cadmium, and nickel) and chlorinated solvents, in the San Francisco Bay Area of northern California. Additional research using models from the Hazardous Air Pollutants program found associations between autism and air toxics at the birth residence of children from North Carolina and West Virginia (Kalkbrenner et al. 2010). Our analysis builds on this work by examining associations with individual-level indicators of exposure based on traffic proximity, prenatally and at birth.

Toxicologic studies suggest a biologically plausible role of air pollution in disrupting brain development and function during critical time points in gestation and early life. Diesel exhaust particles present in traffic-related pollution have been shown to have endocrine-disrupting activity and to transplacentally affect sexual differentiation and alter cognitive function in mice (Hougaard et al. 2008; Watanabe and Kurita 2001). Prenatal exposure to ozone in rats has been seen to alter monoamine content in the cerebellum, which may then alter neural circuitry formation (Gonzalez-Pina et al. 2008). Recent work examining the effects of benzo[*a*]pyrene, a common PAH, indicates that prenatal oral exposure in mice results in decreased neuronal plasticity and behavioral deficits (Brown et al. 2007). Specifically, prenatal exposure was associated with reduced glutamate receptor development when synapses are formed. Additionally, exposure to benzo[*a*]pyrene via breast-feeding in mice during the early postnatal period, corresponding to the rapid human brain development taking place during the third trimester, affected neuromaturation as measured by classic developmental behavior tests and to reduce expression of the serotonin receptor 5HT1A (Bouayed et al. 2009; Pan et al. 2009).

Traffic-related air pollutants have been observed to induce inflammation and oxidative stress after both short-term and long-term exposures in toxicologic and human studies, and these pathways are thought to mediate effects of air pollution on respiratory and cardiovascular disease, and perhaps on neurologic outcomes (Block and Calderon-Garciduenas 2009; Calderon-Garciduenas et al. 2009; Castro-Giner et al. 2009; Gilliland et al. 2004; Künzli et al. 2010). The emerging evidence that oxidative stress and inflammation are also involved in the pathogenesis of autism may suggest a biologically plausible rationale for the observed associations in our study (Boso et al. 2006; Enstrom et al. 2009a, 2009b; James et al. 2004, 2006, 2009). In particular, research examining serum biomarkers reported increased levels of the proinflammatory cytokines tumor necrosis factor-α, interleukin (IL)-6, IL-8, and colony-stimulating factor II, as well as two markers of T-helper 1 immune response (interferon-γ and IL-8), in postmortem brain tissue of autism cases compared with controls (Li et al. 2009). Additional research from the CHARGE study has shown increased plasma levels of immunoglobulin (Ig) G-4 and reduced concentrations of tumor growth factor-β, related to immune response and inflammatory processes, in plasma of children with autism compared with typically developing controls and children with developmental delay (Ashwood et al. 2008; Enstrom et al. 2009a, 2009b). Other recent work indicates that exposure to air pollution exposure during pregnancy is associated with changes in IgE and in lymphocytes measured from cord blood, supporting the idea that maternal exposure to air pollution is associated with altered immune profiles in the fetus (Herr et al. 2010a, 2010b). Moreover, published evidence links maternal antibodies to fetal brain tissue with a subset of autism cases (Braunschweig et al. 2008).

Genetic variation in oxidative stress and inflammatory pathways has also been associated with autism. Oxidative stress endophenotypes and corresponding genotypes related to metabolism of methionine transmethylation and transsulfuration were significantly decreased in children with autism compared with controls, indicating increased susceptibility to oxidative stress (Boso et al. 2006; James et al. 2004, 2006). Markers of lipid peroxidation have also been associated with autism, as have increased levels of nitric oxide and mitochondrial dysfunction, which may be related to the formation of reactive oxygen species (Chauhan and Chauhan 2006; Filipek et al. 2004; Ming et al. 2005; Sogut et al. 2003; Yao et al. 2006). Polymorphisms in glutathione *S*-transferase mu 1 (*GSTM1*), glutathione *S*-transferase pi 1 (*GSTP1*), and glutathione peroxidase 1 (*GPX1*), which modulate the response to oxidative stress, have been associated with increased autism risk (Buyske et al. 2006; Ming et al. 2009; Williams et al. 2007). These genetic variants have also been shown to modify the association between exposure to air oxidant pollutant associations and respiratory outcomes (Islam et al. 2009; Salam et al. 2007). Examination of the interaction between these oxidant-associated genes and environmental exposures may help to clarify susceptibilities to environmental pollutants among children with autism.

We recognize that the moderate relative risks associated with freeway proximity in our study may have been attributable to chance or bias. The study is currently limited by sample size and potential exposure misclassification. Analysis of larger data sets would provide additional valuable insight into these findings and the potential for replication. Although we used a residential history questionnaire (available for a subset of the study participants) to choose the appropriate address for trimester, there still may be misclassification of exposure in these data due to inaccurate date reporting on the part of the mother, or in our choice among multiple addresses in each trimester. We could not distinguish the potential effect of noise from that due to pollutant exposures, both resulting from residential location near a freeway or other road in this study. Addresses on the birth certificate could also be in error, but this would probably be less likely. We were not able to examine specific pollutant concentrations in this study, and the traffic proximity metrics were subject to misclassification of exposure because they did not account for traffic volume or prevailing wind speed and direction. However, this exposure misclassification was unlikely to have been systematically related to disease, and our results may therefore have underestimated the magnitude of a true causal association.

Despite these limitations, this study has several strengths. We assessed autism through well-validated instruments that are recognized as the gold standard in the field. We examined exposure prenatally and at birth, two pivotal times in gestational development, whereas prior work on air pollution has been limited to the birth address or a cumulative lifetime exposure measure. To our knowledge, these results are the first to show an association of autism with residential traffic proximity.

## Conclusions

Little is known about potential environmental contributions to autism. The observed associations with traffic proximity merit further research to determine whether these results are reproducible in populations with improved estimates of exposure to specific ambient air pollutants. Examination of gene–pollution interactions may also help us learn about causal pathways involved in autism and identify potentially susceptible populations and may lead to prevention strategies. Our analysis is the first step in examining a hypothesized relationship between air pollutants and autism. It has been estimated that 11% of the U.S. population lives within 100 m of a four-lane highway, so a causal link to autism or other neurodevelopmental disorders would have broad public health implications (Brugge et al. 2007).

## Figures and Tables

**Table 1:** Demographic characteristics of CHARGE cases with autism and controls with typical development (*n* = 563).

Demographic variable	Percent (*n*)		Chi-square *p*-value
	Cases (*n* = 304)	Controls (*n* = 259)	
Male sex	87 (263)	81 (211)	0.10
Child race/ethnicity			0.21
White	51 (154)	51 (131)	
Hispanic	29 (88)	30 (77)	
Black	3 (8)	2 (5)	
Asian	7 (21)	3 (8)	
Other^a^	11 (33)	15 (38)	
Maximum education in home			0.88
High school or less	7 (22)	8 (22)	
Some college	31 (95)	31 (79)	
Bachelor degree	35 (107)	36 (93)	
Graduate or professional degree	26 (80)	25 (65)	
Maternal smoking during pregnancy^b^	9 (27)	7 (18)	0.40
Maternal age ≥ 35 years	28 (85)	25 (65)	0.44
Preterm delivery (< 259 days)^c^	11 (32)	10 (25)	0.73

a “Other” refers to mixed race/ethnicity or other reported race/ethnicity, including Native American, Indian, East Indian, Cuban, or Mexican American.

b Mother reported smoking at any time during pregnancy.

c Equivalent to 37 completed weeks.

**Table 2:** Exposure ORs (95% CIs) for autism, by category of distance from residence to the nearest freeway at time of birth (*n* = 563).

Exposure category	*n* (cases/controls)	Crude	Adjusted^a^
< 309 m from freeway (closest 10%)	38/19	1.86 (1.04–3.45)	1.86 (1.03–3.45)
309–647 m from freeway (10th to 25th percentile)	43/41	0.98 (0.60–1.59)	0.96 (0.58–1.56)
647–1,419 m from freeway (25th to 50th percentile)	77/63	1.14 (0.76–1.71)	1.11 (0.73–1.67)
> 1,419 m from freeway (further 50%)	146/136	Reference	Reference

a Model was adjusted for child sex (male vs. female), child race/ethnicity (Hispanic vs. white, black/Asian/other vs. white), maximum education of parents (parent with highest of four levels: college degree or higher vs. some high school, high school degree, or some college education), maternal age (> 35 years vs. ≤ 35 years), and maternal smoking during pregnancy (mother reported any smoking during pregnancy vs. mother reported no smoking during pregnancy)

**Table 3:** Exposure ORs (95% CIs) for autism, by category of distance from residence to the nearest major road at time of birth (*n* = 563).

Exposure category	*n* (cases/controls)	Crude	Adjusted^a^
≤ 42 m from major road (closest 10%)	28/30	0.80 (0.45–1.41)	0.71 (0.39–1.26)
42–96 m from major road (10th to 25th percentile)	54/32	1.44 (0.88–2.39)	1.29 (0.77–2.18)
96–209 m from major road (25th to 50th percentile)	71/68	0.89 (0.59–1.34)	0.83 (0.55–1.26)
> 209 m from major road (further 50%)	151/129	Reference	Reference

a Model was adjusted for child sex (male vs. female), child race/ethnicity (Hispanic vs. white, black/Asian/other vs. white), maximum education of parents (parent with the highest of four levels: college degree or more education vs. some high school, high school degree, or some college education), maternal age (> 35 years vs. ≤ 35 years), and maternal smoking during pregnancy (mother reported any smoking during pregnancy vs. mother reported no smoking during pregnancy), and freeway distance categories (< 309 m, 309–647 m, 647–1,419 m vs. referent of > 1,419 m).

## References

[b1-ehp-119-873] Abrahams BS, Geschwind DH (2008). Advances in autism genetics: on the threshold of a new neurobiology. Nat Rev Genet.

[b2-ehp-119-873] American Psychiatric Association (2000). Diagnostic and Statistical Manual of Mental Disorders.

[b3-ehp-119-873] Ashwood P, Enstrom A, Krakowiak P, Hertz-Picciotto I, Hansen RL, Croen LA (2008). Decreased transforming growth factor beta1 in autism: a potential link between immune dysregulation and impairment in clinical behavioral outcomes. J Neuroimmunol.

[b4-ehp-119-873] Block ML, Calderon-Garciduenas L (2009). Air pollution: mechanisms of neuroinflammation and CNS disease. Trends Neurosci.

[b5-ehp-119-873] Bocskay KA, Tang D, Orjuela MA, Liu X, Warburton DP, Perera FP (2005). Chromosomal aberrations in cord blood are associated with prenatal exposure to carcinogenic polycyclic aromatic hydrocarbons. Cancer Epidemiol Biomarkers Prev.

[b6-ehp-119-873] Boso M, Emanuele E, Minoretti P, Arra M, Politi P, Ucelli di Nemi S (2006). Alterations of circulating endogenous secretory RAGE and S100A9 levels indicating dysfunction of the AGE-RAGE axis in autism. Neurosci Lett.

[b7-ehp-119-873] Bouayed J, Desor F, Rammal H, Kiemer AK, Tybl E, Schroeder H (2009). Effects of lactational exposure to benzo[alpha]pyrene (B[alpha]P) on postnatal neurodevelopment, neuronal receptor gene expression and behaviour in mice. Toxicology.

[b8-ehp-119-873] Braunschweig D, Ashwood P, Krakowiak P, Hertz-Picciotto I, Hansen R, Croen LA (2008). Autism: maternally derived antibodies specific for fetal brains. Neurotoxicology.

[b9-ehp-119-873] Brown LA, Khousbouei H, Goodwin JS, Irvin-Wilson CV, Ramesh A, Sheng L (2007). Down-regulation of early ionotrophic glutamate receptor subunit developmental expression as a mechanism for observed plasticity deficits following gestational exposure to benzo(*a*)pyrene. Neurotoxicology.

[b10-ehp-119-873] Brugge D, Durant JL, Rioux C (2007). Near-highway pollutants in motor vehicle exhaust: a review of epidemiologic evidence of cardiac and pulmonary health risks. Environ Health.

[b11-ehp-119-873] Buyske S, Williams TA, Mars AE, Stenroos ES, Ming SX, Wang R (2006). Analysis of case-parent trios at a locus with a deletion allele: association of GSTM1 with autism. BMC Genet.

[b12-ehp-119-873] Calderon-Garciduenas L, Macias-Parra M, Hoffmann HJ, Valencia-Salazar G, Henriquez-Roldan C, Osnaya N (2009). Immunotoxicity and environment: immunodysregulation and systemic inflammation in children. Toxicol Pathol.

[b13-ehp-119-873] Caltrans (2008). Traffic and Vehicle Data Systems Unit: All Traffic Volumes on the California State Highway System.

[b14-ehp-119-873] Castro-Giner F, Künzli N, Jacquemin B, Forsberg B, de Cid R, Sunyer J (2009). Traffic-related air pollution, oxidative stress genes, and asthma (ECHRS). Environ Health Perspect.

[b15-ehp-119-873] Centers for Disease Control and Prevention (2007a). Prevalence of autism spectrum disorders—autism and developmental disabilities monitoring network, 14 sites, United States, 2002. MMWR Surveill Summ.

[b16-ehp-119-873] Centers for Disease Control and Prevention (2007b). Prevalence of autism spectrum disorders—autism and developmental disabilities monitoring network, six sites, United States, 2000. MMWR Surveill Summ.

[b17-ehp-119-873] Centers for Disease Control and Prevention (2009). Prevalence of autism spectrum disorders—autism and developmental disabilities monitoring network, United States, 2006. MMWR Surveill Summ.

[b18-ehp-119-873] Chauhan A, Chauhan V (2006). Oxidative stress in autism. Pathophysiology.

[b19-ehp-119-873] Currie J, Neidell M, Schmieder JF (2009). Air pollution and infant health: lessons from New Jersey. J Health Econ.

[b20-ehp-119-873] Enstrom A, Krakowiak P, Onore C, Pessah IN, Hertz-Picciotto I, Hansen RL (2009a). Increased IgG4 levels in children with autism disorder. Brain Behav Immun.

[b21-ehp-119-873] Enstrom AM, Lit L, Onore CE, Gregg JP, Hansen RL, Pessah IN (2009b). Altered gene expression and function of peripheral blood natural killer cells in children with autism. Brain Behav Immun.

[b22-ehp-119-873] Filipek PA, Juranek J, Nguyen MT, Cummings C, Gargus JJ (2004). Relative carnitine deficiency in autism. J Autism Dev Disord.

[b23-ehp-119-873] Gauderman WJ, Avol E, Lurmann F, Kuenzli N, Gilliland F, Peters J (2005). Childhood asthma and exposure to traffic and nitrogen dioxide. Epidemiology.

[b24-ehp-119-873] Gauderman WJ, Vora H, McConnell R, Berhane K, Gilliland F, Thomas D (2007). Effect of exposure to traffic on lung development from 10 to 18 years of age: a cohort study. Lancet.

[b25-ehp-119-873] Gilliland FD, Li YF, Saxon A, Diaz-Sanchez D (2004). Effect of glutathione-*S*-transferase M1 and P1 genotypes on xenobiotic enhancement of allergic responses: randomised, placebo-controlled crossover study. Lancet.

[b26-ehp-119-873] Gonzalez-Pina R, Escalante-Membrillo C, Alfaro-Rodriguez A, Gonzalez-Maciel A (2008). Prenatal exposure to ozone disrupts cerebellar monoamine contents in newborn rats. Neurochem Res.

[b27-ehp-119-873] Hansen CA, Barnett AG, Pritchard G (2008). The effect of ambient air pollution during early pregnancy on fetal ultrasonic measurements during mid-pregnancy. Environ Health Perspect.

[b28-ehp-119-873] Herr CEW, Dostal M, Ghosh R, Ashwood P, Lipsett M, Pinkerton KE (2010b). Air pollution exposure during critical time periods in gestation and alterations in cord blood lymphocyte distribution: a cohort of livebirths. Environ Health.

[b29-ehp-119-873] Herr CEW, Ghosh R, Dostal M, Skokanova V, Ashwood P, Lipsett M (2010a). Exposure to air pollution in critical prenatal time windows and IgE levels in newborns. Pediatr Allergy Immunol.

[b30-ehp-119-873] Hertz-Picciotto I, Croen LA, Hansen R, Jones CR, van de Water J, Pessah IN (2006). The CHARGE study: an epidemiologic investigation of genetic and environmental factors contributing to autism. Environ Health Perspect.

[b31-ehp-119-873] Hertz-Picciotto I, Delwiche L (2009). The rise in autism and the role of age at diagnosis. Epidemiology.

[b32-ehp-119-873] Hougaard KS, Jensen KA, Nordly P, Taxvig C, Vogel U, Saber AT (2008). Effects of prenatal exposure to diesel exhaust particles on postnatal development, behavior, genotoxicity and inflammation in mice. Part Fibre Toxicol.

[b33-ehp-119-873] Islam T, Berhane K, McConnell R, Gauderman WJ, Avol E, Peters JM (2009). Glutathione-*S*-transferase (GST) P1, GSTM1, exercise, ozone and asthma incidence in school children. Thorax.

[b34-ehp-119-873] James SJ, Cutler P, Melnyk S, Jernigan S, Janak L, Gaylor DW (2004). Metabolic biomarkers of increased oxidative stress and impaired methylation capacity in children with autism. Am J Clin Nutr.

[b35-ehp-119-873] James SJ, Melnyk S, Jernigan S, Cleves MA, Halsted CH, Wong DH (2006). Metabolic endophenotype and related genotypes are associated with oxidative stress in children with autism. Am J Med Genet B Neuropsychiatr Genet.

[b36-ehp-119-873] James SJ, Rose S, Melnyk S, Jernigan S, Blossom S, Pavliv O (2009). Cellular and mitochondrial glutathione redox imbalance in lymphoblastoid cells derived from children with autism. FASEB J.

[b37-ehp-119-873] Kalkbrenner AE, Daniels JL, Chen JC, Poole C, Emch M, Morrissey J (2010). Perinatal exposure to hazardous air pollutants and autism spectrum disorders at age 8. Epidemiology.

[b38-ehp-119-873] King M, Bearman P (2009). Diagnostic change and the increased prevalence of autism. Int J Epidemiol.

[b39-ehp-119-873] Künzli N, Jerrett M, Garcia-Esteban R, Basagana X, Beckermann B, Gilliland F (2010). Ambient air pollution and the progression of atherosclerosis in adults. PLoS One.

[b40-ehp-119-873] Le Couteur A, Lord C, Rutter M (2003). Autism Diagnostic Interview–Revised (ADI-R).

[b41-ehp-119-873] Li X, Chauhan A, Sheikh AM, Patil S, Chauhan V, Li XM (2009). Elevated immune response in the brain of autistic patients. J Neuroimmunol.

[b42-ehp-119-873] Lord C, Rutter M, DiLavore P, Risi S (2003). Autism Diagnostic Observation Schedule Manual.

[b43-ehp-119-873] Ma D, Salyakina D, Jaworski JM, Konidari I, Whitehead PL, Andersen AN (2009). A genome-wide association study of autism reveals a common novel risk locus at 5p14.1. Ann Hum Genet.

[b44-ehp-119-873] McConnell R, Berhane K, Yao L, Jerrett M, Lurmann F, Gilliland F (2006). Traffic, susceptibility, and childhood asthma. Environ Health Perspect.

[b45-ehp-119-873] McConnell R, Islam T, Shankardass K, Jerrett M, Lurmann F, Gilliland F (2010). Childhood incident asthma and traffic-related air pollution at home and school. Environ Health Perspect.

[b46-ehp-119-873] Ming X, Johnson WG, Stenroos ES, Mars A, Lambert GH, Buyske S (2009). Genetic variant of glutathione peroxidase 1 in autism. Brain Dev.

[b47-ehp-119-873] Ming X, Stein TP, Brimacombe M, Johnson WG, Lambert GH, Wagner GC (2005). Increased excretion of a lipid peroxidation biomarker in autism. Prostaglandins Leukot Essent Fatty Acids.

[b48-ehp-119-873] Morales E, Julvez J, Torrent M, de Cid R, Guxens M, Bustamante M (2009). Association of early-life exposure to household gas appliances and indoor nitrogen dioxide with cognition and attention behavior in preschoolers. Am J Epidemiol.

[b49-ehp-119-873] Muhle R, Trentacoste SV, Rapin I (2004). The genetics of autism. Pediatrics.

[b50-ehp-119-873] Mullen E (1995). Mullen Scales of Early Learning.

[b51-ehp-119-873] Pan IJ, Daniels JL, Goldman BD, Herring AH, Siega-Riz AM, Rogan WJ (2009). Lactational exposure to polychlorinated biphenyls, dichlorodiphenyltrichloroethane, and dichlorodiphenyldichloroethylene and infant neurodevelopment: an analysis of the pregnancy, infection, and nutrition babies study. Environ Health Perspect.

[b52-ehp-119-873] Perera FP, Rauh V, Tsai WY, Kinney P, Camann D, Barr D (2003). Effects of transplacental exposure to environmental pollutants on birth outcomes in a multiethnic population. Environ Health Perspect.

[b53-ehp-119-873] Perera FP, Rauh V, Whyatt RM, Tsai WY, Bernert JT, Tu YH (2004). Molecular evidence of an interaction between prenatal environmental exposures and birth outcomes in a multiethnic population. Environ Health Perspect.

[b54-ehp-119-873] Perera FP, Rauh V, Whyatt RM, Tsai WY, Tang D, Diaz D (2006). Effect of prenatal exposure to airborne polycyclic aromatic hydrocarbons on neurodevelopment in the first 3 years of life among inner-city children. Environ Health Perspect.

[b55-ehp-119-873] Perera FP, Tang D, Rauh V, Tu YH, Tsai WY, Becker M (2007). Relationship between polycyclic aromatic hydrocarbon–DNA adducts, environmental tobacco smoke, and child development in the World Trade Center cohort. Environ Health Perspect.

[b56-ehp-119-873] Ritz B, Yu F (1999). The effect of ambient carbon monoxide on low birth weight among children born in Southern California between 1989 and 1993. Environ Health Perspect.

[b57-ehp-119-873] Rutter M, Bailey A, Lord C (2003). A Social Communication Questionnaire (SCQ).

[b58-ehp-119-873] Salam MT, Lin PC, Avol EL, Gauderman WJ, Gilliland FD (2007). Microsomal epoxide hydrolase, glutathione *S*-transferase P1, traffic and childhood asthma. Thorax.

[b59-ehp-119-873] Sexton K, Gong H, Bailar JC, Ford JG, Gold DR, Lambert WE (1993). Air pollution health risks: do class and race matter?. Toxicol Ind Health.

[b60-ehp-119-873] Sogut S, Zoroglu SS, Ozyurt H, Yilmaz HR, Ozugurlu F, Sivasli E (2003). Changes in nitric oxide levels and antioxidant enzyme activities may have a role in the pathophysiological mechanisms involved in autism. Clin Chim Acta.

[b61-ehp-119-873] Sparrow S, Cicchettim D, Balla D (1984). Vineland Adaptive Behavior Scales Interview Edition Expanded Form Manual.

[b62-ehp-119-873] Suglia SF, Gryparis A, Wright RO, Schwartz J, Wright RJ (2008). Association of black carbon with cognition among children in a prospective birth cohort study. Am J Epidemiol.

[b63-ehp-119-873] Van Meter KC, Christiansen LE, Delwiche LD, Azari R, Carpenter TE, Hertz-Picciotto I (2010). Geographic distribution of autism in California: a retrospective birth cohort analysis. Autism Res.

[b64-ehp-119-873] Vassilev ZP, Robson MG, Klotz JB (2001). Outdoor exposure to airborne polycyclic organic matter and adverse reproductive outcomes: a pilot study. Am J Ind Med.

[b65-ehp-119-873] Wang K, Zhang H, Ma D, Bucan M, Glessner JT, Abrahams BS (2009). Common genetic variants on 5p14.1 associate with autism spectrum disorders. Nature.

[b66-ehp-119-873] Watanabe N, Kurita M (2001). The masculinization of the fetus during pregnancy due to inhalation of diesel exhaust. Environ Health Perspect.

[b67-ehp-119-873] Williams TA, Mars AE, Buyske SG, Stenroos ES, Wang R, Factura-Santiago MF (2007). Risk of autistic disorder in affected offspring of mothers with a glutathione *S*-transferase P1 haplotype. Arch Pediatr Adolesc Med.

[b68-ehp-119-873] Windham GC, Zhang L, Gunier R, Croen LA, Grether JK (2006). Autism spectrum disorders in relation to distribution of hazardous air pollutants in the San Francisco Bay Area. Environ Health Perspect.

[b69-ehp-119-873] Yao Y, Walsh WJ, McGinnis WR, Pratico D (2006). Altered vascular phenotype in autism: correlation with oxidative stress. Arch Neurol.

[b70-ehp-119-873] Zhu Y, Hinds WC, Kim S, Sioutas C (2002). Concentration and size distribution of ultrafine particles near a major highway. J Air Waste Manag Assoc.

[b71-ehp-119-873] Zhu Y, Kuhn T, Mayo P, Hinds WC (2006). Comparison of daytime and nighttime concentration profiles and size distributions of ultrafine particles near a major highway. Environ Sci Technol.

